# Enhancing Post‐Discharge Care for People Who Have Had an Acute Myocardial Infarction in Portugal: Insights From Patient Journey Mapping

**DOI:** 10.1111/hex.70521

**Published:** 2025-12-19

**Authors:** Filipa Homem, Andreia Gomes, Helena Martins, Ana Caetano, Maurício Alves, Luís Rodrigues, Mariana Simões, Sofia Martinho, Sílvia Monteiro, Catarina Veiga, Emília Correia, Dário Lemos, Verónica Coutinho, António Amaral

**Affiliations:** ^1^ Coimbra University, UICISA:E/ESEnfC, ULS Coimbra Coimbra Portugal; ^2^ ULS Coimbra, UICISA:E/ESEnfC Coimbra Portugal; ^3^ Portuguese National Health Service Executive Board, UICISA:E/ESEnfC Coimbra Portugal; ^4^ UICISA:E/ESEnfC Coimbra Portugal

**Keywords:** health journey, myocardial infarction, patient experience, patient journey mapping

## Abstract

**Introduction:**

Cardiovascular disease is the leading cause of death and disability, with the post‐discharge period being particularly vulnerable for patients due to communication issues, medication errors, and inadequate follow‐up. This study explores the patient experience following acute myocardial infarction in the Portuguese healthcare system, providing insights to improve care quality, outcomes, and satisfaction.

**Methods:**

This study used qualitative patient journey mapping through semi‐structured telephone interviews with people who have had an acute myocardial infarction discharged between January and December 2023, to explore their experiences, identify areas for improvement in the Portuguese healthcare system, and develop patient‐centred solutions. To complement self‐reported data, data were collected from patients’ electronic health records.

**Results:**

While participants were generally satisfied with the care provided during hospitalisation and discharge, significant gaps were identified, such as no shared decision‐making on goal setting, limited medication counselling, insufficient coordination with Primary Health Care (PHC), and delays in hospital follow‐up beyond European Society of Cardiology guideline recommendations. Many patients had limited follow‐up in PHC, with follow‐up appointments often relying on patient initiative, and long waiting times for hospital follow‐up care. By combining patient narratives with electronic health records and multidisciplinary validation, the findings underscore the need for a more integrated and patient‐centred approach.

**Conclusions:**

Post‐acute myocardial infarction care in Portugal shows critical gaps in discharge planning, PHC coordination, and timely follow‐up. Strengthening patient‐centred strategies, including structured protocols, systematic use of Patient‐Reported Outcome and Experience Measures, earlier and nurse‐led PHC follow‐up, and stronger coordination across care levels, are essential to enhance recovery, reduce readmissions, and improve long‐term outcomes.

**Patient or Public Contribution:**

Data were analysed by a multidisciplinary team, which included two patient representatives with lived experience of myocardial infarction, to ensure that the findings reflect both clinical perspectives and patient priorities.

**Abbreviations**
AMI: acute myocardial infarction
CRP: cardiac rehabilitation programme
ESC: European Society of Cardiology
IQR: interquartile range
OECD: Organisation for Economic Co‐operation and Development
P: patient
PHC: primary health care
PJM: patient journey mapping
PREM: patient‐reported experience measure
PROM: patient‐reported outcome measure
RSE: Portuguese Electronic Health Records (in Portuguese: Registo de Saúde Eletrónico)
SDM: shared decision‐making
SNS: Portuguese National Health Service (in Portuguese: Serviço Nacional de Saúde)
ULS: Portuguese Local Health Unit (in Portuguese: Unidade Local de Saúde)

## 1 Introduction

Cardiovascular disease (CVD) remains the leading cause of death worldwide and a significant contributor to disability. With ageing populations and improved survival rates following initial cardiac events, the burden of disability‐adjusted life years is increasing, placing considerable pressure on healthcare systems [1]. In this context of high demand and limited resources, patients with CVD often move through complex care pathways, engaging in only brief interactions with multiple healthcare professionals [2].

While primary prevention and treatment of acute myocardial infarction (AMI) are well‐established, considerable challenges persist in secondary prevention. This approach is most effective when it integrates regular clinical follow‐up with structured monitoring, lifestyle counselling, adherence to pharmacological therapy, and participation in cardiac rehabilitation programmes (CRP). Such a comprehensive strategy not only reduces mortality and the risk of recurrent infarction but also lowers hospital readmissions and emergency visits, while improving patients' overall quality of life [3].

The immediate post‐discharge period is a particularly vulnerable time for patients, as the transition from inpatient to outpatient care is precarious. Those who are clinically most vulnerable at discharge face heightened risks, often exacerbated by deficiencies in transitional care [2]. Communication failures, inadequate medication counselling, absence of shared treatment goals, and insufficient follow‐up have been linked to preventable readmissions and poor long‐term outcomes [4, 5]. Despite these risks, transitional care practices remain highly variable between and even within healthcare systems, leading to inconsistent patient support [6, 7].

Transitional care interventions, such as structured discharge planning, follow‐up calls and shared decision‐making, can improve patient outcomes by reducing Emergency Department visits, unplanned 30‐day readmissions, and mortality following AMI, while also enhancing satisfaction [2, 3, 6]. Yet, evidence from Southern Europe remains limited. Considering the marked differences in healthcare expenditure, procedural rates, lifestyle factors, and resource availability between Southern and Western Europe, patient experiences and unmet needs are likely to diverge substantially [8]. Moreover, while most existing literature emphasises clinical outcomes, far less attention has been given to patients’ lived experiences, preferences and barriers to accessing timely and continuous care. Capturing these perspectives is essential for designing patient‐centred integrated care models [9], as emphasised in recent European Society of Cardiology (ESC) guidelines [3].

Little is known about how patients in Portugal experience the transition of care following AMI. Recently, the study ‘Post‐myocardial infarction patient pathways in Portugal’ provided valuable insights but focused exclusively on cardiologists and general practitioners, leaving patients’ voices unheard [10].

This study aims to address these gaps by exploring the patient experience following AMI within the Portuguese healthcare system, using patient journey mapping. By capturing patients’ challenges, touchpoints, and priorities, the study seeks to generate actionable insights to strengthen continuity of care and to inform the development of patient‐centred solutions aligned with the broader goals of integrated and value‐based care.

## 2 Methods

### 2.1 Study Design

Patient journey mapping (PJM) is an emerging qualitative method that provides a structured approach to understand patients' experiences across different points of care. It has been successfully applied in various healthcare settings to identify system gaps, optimise service design, and enhance communication between patients and providers [11, 12]. Despite its demonstrated value, its application in cardiovascular care, particularly in transitional care following AMI, remains limited [13, 14].

Addressing this void, the present study represents the first phase of the Cardiac Integrated Care project, a participatory health initiative aimed at improving care transitions and promoting self‐care after AMI. A multidisciplinary research team was assembled, including nurses from cardiology and PHC, cardiologists, general practitioners, pharmacists and patient representatives.

Through PJM, the study explored individuals’ experiences of care within the Portuguese healthcare system, providing a patient‐centred lens on how individuals interact with healthcare services over time. This approach not only identifies gaps and challenges but also highlights opportunities for targeted improvements in care delivery and patient engagement [11, 12].

### 2.2 Setting

The Portuguese National Health Service (in Portuguese – SNS) provides universal healthcare coverage in Portugal, ensuring that all residents have access to medical care, including primary, secondary and tertiary services. It is publicly funded through taxation and recently organised into Local Health Units (in Portuguese – ULS) that coordinate hospitals, PHC and specialised services. The SNS emphasises equitable access, preventive care, and integrated management of chronic and acute conditions, guiding patient referrals according to clinical needs and facility capabilities [15].

In Portugal, hospitals are classified according to their level of specialisation and support capabilities. Regarding cardiology services, type A hospitals correspond to tertiary centres, offering comprehensive cardiology services, including a cardiology intensive care unit, a permanently available hemodynamic laboratory, and cardiac surgery. Type B2 hospitals provide advanced support, with a cardiology intensive care unit and a hemodynamic laboratory, but without cardiac surgery. Type B1 hospitals offer outpatient and inpatient cardiology consultations and are equipped with noninvasive diagnostic tools. This classification helps guide patient referral, ensuring that critical cases, such as AMI, are treated promptly in facilities with the appropriate resources and expertise [16].

All healthcare professionals in Portugal have access to patients’ electronic health records across all levels of care through the Shared Health Record System (in Portuguese – RSE). This platform allows secure, real‐time sharing of clinical information between primary, secondary, and tertiary care providers. Additionally, patients can access health information and services through the SNS24 app, which facilitates communication with healthcare professionals and appointment scheduling. This recent electronic system is continuously evolving. Only now allows notifications to PHC upon patient discharge, improving continuity of care. At the same time, patients are beginning to use the SNS24 app for accessing health information and communicating with healthcare professionals, marking the first steps toward more integrated digital health management. Effective implementation, however, requires proper training for healthcare staff to ensure the system is used correctly and efficiently [17].

### 2.3 Research Material Development

Several research materials were developed specifically for this study. First, an interview guide was designed based on a PJM framework [12] to capture patients' lived experiences across different stages of their care pathway, and to identify key touchpoints, barriers and facilitators (supplementary data available upon request). The semi‐structured guide was organised along the typical post‐AMI pathway: (i) Preadmission engagement with PHC, (ii) hospital discharge, (iii) PHC follow‐up and (iv) hospital follow‐up. For each stage, open‐ended questions were formulated to elicit participants' experiences, perceptions, and unmet needs (e.g., ‘When you leave the hospital, do you feel you have a clear understanding of your health condition and of the factors that may improve or worsen it?’; ‘Did you feel confident upon discharge that you were able to manage your health?’; ‘What challenges did you experience?’). Probes were informed by core PJM concepts such as communication, continuity, coordination, and shared treatment goals, ensuring comparability across interviews while leaving space for individual narratives.

In addition to qualitative exploration, quantitative measures were also embedded within the interviews. At the end of each interview, participants were asked to rate their overall satisfaction with the care received and whether they would recommend the services to family or friends, using a numeric rating scale from 0 to 10 (0 = not at all satisfied; 10 = extremely satisfied) [18]. These ratings provided a standardised measure of satisfaction, allowing descriptive statistical reporting. Satisfaction with hospital follow‐up, however, was not assessed separately, as this stage occurred within the same institutional setting as the initial hospitalisation and discharge. The research team determined that distinguishing between satisfaction with discharge and hospital follow‐up could lead to conceptual overlap and respondent confusion, given that both experiences were part of a continuous hospital‐based care process.

To complement self‐reported data, a data extraction grid was developed to collect information from patients' electronic health records, including age, sex, hospital stay duration, documented cardiovascular risk factors, discharge letter and discharge summaries, dates of PHC and hospital consultations before and after the AMI event, and readmissions. This allowed triangulation and validation of patient‐reported information.

Finally, standard research materials were also produced in line with institutional ethical requirements. All instruments were reviewed and validated by the research team before their use.

### 2.4 Participant Recruitment

Patients aged 18 years or older who were diagnosed with AMI and discharged between January and December 2023 from a type A hospital were eligible for inclusion. Participants were identified through electronic health records. To ensure continuity of care and facilitate follow‐up, only those currently registered within the same Local Health Unit (ULS) as the discharging hospital and with a valid telephone contact in their electronic record were considered eligible.

Recruitment took place between February and April 2024. Eligible patients were first identified by the hospital's cardiology department, and their contact details were securely shared with the research team in accordance with institutional data protection procedures. The lead researcher then contacted each potential participant by telephone, following a standardised recruitment script.

During this call, patients were verbally informed about the study's objectives, voluntary nature, expected duration, confidentiality assurances, and their right to withdraw at any time without any impact on their medical care. No written participant information sheet was provided due to the telephone‐based design of the study. Instead, all relevant information was explained verbally during the call, and participants were invited to ask any questions before deciding whether to participate. Verbal informed consent was obtained and recorded in a secure study log before the start of the interview. This consent covered both participation in the interview and authorisation to access relevant clinical data from the electronic health record.

For methodological clarity, we adopted a staged purposive sampling strategy. We began with younger patients, typically with fewer comorbidities and more linear care trajectories, to establish a clear, foundational comprehension of key journey stages. This ‘simpler‐case‐first’ approach aligns with purposive and theoretical sampling principles (initiating analysis with the most information‐rich cases and then expanding to more complex ones as coding evolves) [19, 20]. Moreover, by sequencing interviews strategically, we enhanced our ability to monitor thematic saturation and, in line with saturation methodology, confirm whether later data added new insights [21].

### 2.5 Data Collection

All interviews were conducted by a cardiology nurse, the lead researcher, with no direct care to any of the participants. At the start of each interview, patients were informed that participation was entirely voluntary and that their responses, or decision not to participate, would not affect their current or future care in any way. Participants were also informed that their input could contribute to potential improvements in the national healthcare system, and that they should provide honest responses based on their experiences, rather than answering in a way they thought might please healthcare professionals.

Semi‐structured telephone interviews were conducted between 2 April and 24 May 2024. The interview guide included open‐ended questions focused on the patient's experience from hospital admission through post‐discharge care, including perceived quality of discharge planning, medication education, follow‐up in primary and secondary care, and goal‐setting practices. Telephone interviews were used due to geographic dispersion and to ensure the inclusion of patients who may not be able to attend face‐to‐face sessions. While telephone‐based methods limit the visual and participatory nature of traditional journey mapping, the approach was adapted to focus on rich narrative data, which served as the foundation for constructing the journey map.

Each interview lasted approximately 22 min and was audio‐recorded using a secure, password‐protected digital recorder.

Data saturation was reached after the 17th interview, when no new codes or themes emerged from the analysis. Two additional interviews were conducted thereafter to confirm saturation and ensure robustness of the findings, resulting in a final sample of 19 participants.

Demographic and clinical data (age, sex, hospital stay duration, readmissions, cardiovascular risk factors, discharge letter and summaries, and dates of PHC and hospital consultations) were extracted from electronic health records following interview completion, with patient consent.

### 2.6 Data Analysis

#### 2.6.1 PJM Interviews

The interviews were audio recorded and transcribed verbatim in Microsoft Word^©^ by the lead researcher, who also conducted the interviews. Transcriptions were manually reviewed and corrected by two additional researchers to ensure accuracy, anonymisation and contextual sensitivity and fidelity to patients' language and tone. An iterative thematic analysis was conducted using both inductive and deductive coding strategies. Three members of the research team independently read all interview transcripts to achieve familiarisation and gain an in‐depth understanding of participants' experiences. Initial inductive coding was carried out collaboratively by two researchers with backgrounds in qualitative research and health services, who identified meaningful excerpts that reflected patients' accounts of experiences, needs, and challenges at different points of care. Emerging codes were compared, discussed, and refined in regular team meetings to ensure consistency. These codes were then grouped into broader categories and organised into overarching themes and touchpoints across the continuum of care. Coding was supported using NVivo® (version 14) qualitative data analysis software. This iterative process allowed codes to evolve based on emerging insights and ensured a transparent and systematic rigour.

Building on this inductive phase, patient narratives were synthesised into a visual journey map using a structured mapping framework adapted from Bulto [12]. This step represents the deductive dimension of the analysis, as it applied an established model to organise experiences chronologically, highlighting critical transitions, system gaps, and opportunities for improvement. Although participants did not cocreate the visual map in real time (due to the telephone format), their detailed descriptions formed the basis of the map's structure. To address the lack of visual interaction, the interviewer used prompts to encourage patients to describe the sequence of events and specific service interactions in detail.

To ensure accuracy, relevance, and patient‐centeredness, the preliminary journey map was then reviewed collaboratively by the multidisciplinary research team (*n* = 12), including two patient representatives. The qualitative researchers led the synthesis, while the remaining team members, clinicians and patient representatives validated the thematic interpretation, ensuring that the final map faithfully reflected patients' lived experiences from both emotional and procedural perspectives [22].

#### 2.6.2 Health Record Data

Patient demographics, pre‐existing cardiovascular risk factors, hospital readmissions and dates of PHC and hospital follow‐up were derived from both self‐reports and electronic health records. This information, alongside discharge letters and summaries, served as triangulation sources to complement interview data and increase reliability. When discrepancies arose between patient self‐reports and health records, a consensus‐based decision process was followed, with health records considered the more reliable source, particularly in cases where patients reported not having a specific condition despite being prescribed related medication [23]. This approach ensured consistency and minimised the risk of underreporting of cardiovascular risk factors.

Discharge summaries were compared with patient‐reported information to identify gaps between clinical documentation and patient understanding. Finally, all qualitative and quantitative findings were systematically compared with the ESC guidelines to identify deviations from recommended care, highlight unmet patient needs, and pinpoint areas for potential improvement in line with established best practices.

Quantitative data from electronic health records were analysed using descriptive statistics to summarise the sample characteristics. Given the small sample size (*n* = 19) and the non‐normal distribution of some variables, medians and interquartile ranges (IQRs) were reported for continuous variables (e.g., age, waiting times, and length of hospital stay) to better represent central tendency and variability, while frequencies and percentages were used for categorical data (e.g., sex, comorbidities, and follow‐up occurrence). For ordinal variables such as satisfaction ratings, the median was used as the primary measure of central tendency, complemented by the interquartile range.

### 2.7 Ethics

Ethical approval was obtained from the Ethics Committee on 8 March 2024 (2024‐1‐ESI. SF). The participation was voluntary, and those who agreed provided verbal informed consent during telephone interviews.

## 3 Results

Of the 76 eligible patients, 19 were recruited for the study, comprising 14 (73.7%) men and 5 (26.3%) women. Participants’ ages ranged from 38 to 65 years, with a median age of 55 years (IQR: 50–59).

The time of hospital stay of all participants ranged between 1 and 12 days, with a median of 3 days (IQR: 3–4.5). Seven (36.8%) participants were not discharged directly from the cardiology service. Instead, they were transferred either to the cardiothoracic surgery service (*n* = 5) or to the internal medicine service (*n* = 2) within the same hospital. For five (26.3%) participants, this hospital admission was not their first, as they had a prior history of coronary artery disease. Following discharge, there were four (21.1%) hospital readmissions: one within 30 days and one within 90 days, both due to decompensated heart failure; one within 4 months, related to chest pain after discontinuation of AMI medication; and one within 8 months, due to rectal bleeding associated with AMI medication.

Data analysis identified four key themes that reflect participants’ experiences across critical points of care within the Portuguese healthcare system: (i) preadmission engagement with PHC, (ii) hospital discharge, (iii) PHC follow‐up and (iv) hospital follow‐up. Across these stages, three cross‐cutting subthemes consistently emerged (1): reactive rather than proactive care (2); fragmented communication and unclear accountability, and (3) limited patient engagement and shared decision‐making. These subthemes weave through the entire patient journey, influencing perceptions of care quality and continuity.

### 3.1 Preadmission Engagement With Primary Healthcare Centres (PHC)

Analysis of pre‐existing cardiovascular risk factors before hospitalisation for AMI, Table 1 shows that most participants had conditions such as dyslipidemia (63.2%), hypertension (42.1%) and/or diabetes (31.6%). However, discrepancies were observed between self‐reported information and electronic health records from PHC and hospital.

**Table 1:** Pre‐existing cardiovascular risk factors.

	Self‐reported		Health records			
			PHC		Hospital	
	*N*	%	*N*	%	*N*	%
Dyslipidemia	2	10.5	7	36.8	12	63.2
Hypertension	4	21.1	8	42.1	8	42.1
Diabetes	4	21.1	6	31.6	6	31.6
Coronary disease	5	26.3	3	15.8	5	26.3
Smoking	4	21.1	1	5.3	4	21.1
Obstructive sleep apnoea	0	0	1	5.3	2	10.5
Depression	1	5.3	1	5.3	1	5.3

Certain diagnoses, particularly smoking and, to a lesser extent, coronary disease, appeared underreported in PHC health records. For example, while four (21.1%) participants self‐identified as smokers, only one (5.3%) had this diagnosis documented in PHC. In the case of dyslipidemia, it was self‐reported by only two participants but recorded in seven PHC records and in 12 hospital records, suggesting underdiagnosis. In contrast, risk factors such as hypertension and diabetes were more consistently documented in PHC records, often at higher rates than self‐reported, suggesting possible gaps in patients' awareness of their conditions or in the communication of diagnoses. Overall, smoking and dyslipidemia stand out as a markedly under‐identified risk factor in PHC.

In terms of preventive care, only seven (36.8%) participants reported regular follow‐up in PHC before their AMI, and these were mainly individuals with diabetes and/or hypertension [‘I am regularly followed by my GP’ (P16)]. Among the five (26.3%) participants with a history of coronary disease, just one reported close cardiovascular risk management in PHC.

Engagement with PHC was generally described as sporadic and reactive, often limited to acute problems or administrative needs: ‘only when I'm sick’ (P2), ‘just for glasses and renewing my driver's license’ (P17), ‘only for vaccines’ (P19), or ‘only if there's no other option because I don't even know my GP…’ (P15).

In summary, participants' experiences before hospitalisation revealed a pattern of reactive rather than proactive care, with PHC contact largely initiated by patients in response to acute issues or administrative needs rather than structured prevention. This reactive approach was compounded by fragmented communication and unclear accountability between care providers, as reflected in discrepancies between self‐reported conditions and PHC records, particularly for smoking and dyslipidemia, suggesting gaps in information sharing and follow‐up responsibility. Furthermore, limited patient engagement and shared decision‐making were evident in the absence of proactive risk monitoring or collaborative goal setting, even among those with known coronary disease, underscoring missed opportunities for early intervention and continuity of care.

### 3.2 Hospital Discharge

Participants expressed a high level of satisfaction with in‐hospital care and discharge planning, rating services between 8 and 10, with a median of 10 (IQR: 9.5–10). One participant said, ‘Many people criticise hospitals, but I was very well treated. And when I have any doubts, I send an email or call my cardiologist’ (P6). One participant expressed their deep appreciation, stating, ‘Excuse my emotion, but I don't have words to describe how I was treated’ (P12).

Overall, while satisfaction was high in terms of discharge quality and the care provided during hospitalisation, some areas for improvement were identified.

When asked about treatment goals at discharge, 11 (57.9%) participants recalled receiving some form of guidance, generally broad and related to lifestyle change or medication adherence. Examples included advice about managing cholesterol, blood glucose, or international normalised ratio (INR) levels, along with general recommendations about diet or physical activity. For instance, one participant explained, ‘Yes, related to cholesterol, blood glucose, and INR’ (P1). Another recalled being told to ‘take the medication and follow a diet’ (P19). A few participants mentioned advice linked to exercise or smoking cessation, such as being encouraged to walk, cycle or swim: ‘They told me to exercise… swimming since I have knee prostheses… but I haven't signed up yet, I admit’ (P16).

However, participants emphasised that these recommendations were rarely translated into measurable or time‐bound targets. Three (15.8%) could identify a concrete behavioural expectation, such as quitting smoking. Most reported that specific targets, such as weight reduction or exercise frequency, were not discussed. As one participant noted: ‘The levels of things were not mentioned; they just told me I had to quit smoking’ (P14). This lack of specificity often left patients with a general sense of what they should do, but without clear parameters for success.

Others denied receiving any recommendations at all, with one participant explaining: ‘I was given a letter with the prescriptions, but absolutely nothing was said… Then I asked why I had a heart attack. She replied… because LDL cholesterol was very high… I set my own goals’ (P17).

Document analysis of discharge letters reinforced these findings. Recommendations were generally phrased in broad terms, such as ‘take medication,’ ‘maintain a balanced diet,’ and ‘engage in physical activity’, without measurable targets.

Notably, only eight (42.1%) participants reported reading their discharge letters. Most handed them unopened to their GP, sometimes because they believed (rightly or wrongly) that the letters should remain sealed: ‘I don't even open the letter. I can't hand an open letter to the GP. And even if I did, I wouldn't understand anything that's written inside’ (P8). Whether this reflects actual practice at PHC or patient misunderstanding remains unclear, but in either case, it represents a barrier to patient engagement with their care plan.

Sixteen (84.2%) participants reported that medication regimens were explained at discharge, although the level of detail and clarity varied. Both interviews and discharge letters confirmed that potential side effects were rarely addressed, representing a consistent gap in communication.

While some participants described efforts by healthcare professionals to clarify medication use, sometimes involving family members, these explanations were generally focused on dosage and administration rather than risks or precautions. For example, one participant noted, ‘They explained everything clearly with my daughter because I am very forgetful’ (P8). Another said, ‘The nurses would explain everything to me when I had doubts about the medication’ (P6). This suggests that while patients and families could obtain clarification when actively seeking it, side effects were not routinely discussed unless prompted. The lack of proactive information about risks may reduce patients’ ability to anticipate and manage adverse effects.

The absence of proactive information about risks may hinder patients’ ability to anticipate and manage adverse effects, ultimately constraining both self‐management and adherence. As one participant emphasised: ‘They should better explain the medications, what they do, and their side effects. Understanding people's health literacy and addressing that would be helpful’ (P17).

Overall, the hospital discharge experience reflected a pattern of reactive rather than proactive care, in which information was provided at the end of hospitalisation but without structured follow‐up or personalised goal setting to support long‐term recovery. Communication across care levels appeared fragmented and marked by unclear accountability, as discharge letters, often written in broad, technical terms, were inconsistently read or understood by patients and not always effectively integrated into PHC records or actions. This lack of coordination limited continuity of care and left patients uncertain about next steps after leaving the hospital. Furthermore, limited patient engagement and shared decision‐making characterised most discharge interactions: treatment goals were delivered rather than co‐created, and while medication adherence was emphasised, patients rarely received tailored explanations or discussions about side effects, self‐management, or individualised behavioural targets. Together, these elements underscore a discharge process centred on information delivery rather than empowerment, reducing opportunities for sustained engagement and preventive care.

### 3.3 PHC Follow‐Up

Overall satisfaction with PHC was moderate, rating services between 4 and 10, with a median of 8 (IQR: 7.5–10). Participants generally perceived PHC as of lower quality compared to hospital care. As onde remarked, ‘I trust my GP, but the PHC… I can't ask too much because there's not much they can do!’ (P16). Another suggested the need for more proactive engagement: ‘There should be more effective and closer follow‐up with patients to prevent relapses’ (P1).

The majority of participants (73.7%) visited the PHC to hand over their discharge notes. However, a few participants (26.3%) did not deliver their notes, either because they perceived it as unnecessary or had negative past experiences with their GP. For example, one participant recalled, ‘I never went there because I once had a GP who, when I handed over letters from the oncology hospital, said… ‘Why would I need that?’ So I stopped delivering them!’ (P15).

Regarding follow‐up, 16 (84.2%) participants stated they were not invited to appointments by the PHC. Instead, most had to take the initiative themselves: ‘I wasn't invited. I scheduled it myself’ (P16). In some cases, GPs considered follow‐up unnecessary when hospital care was ongoing: ‘Since the doctor knew I was being followed up at the hospital, she (GP) didn't need to schedule an appointment’ (P12).

When questioned about follow‐up at PHC after discharge, 8 (42.1%) participants reported receiving no follow‐up, while 11 (57.9%) reported some form of follow‐up. Among these, eight had their first PHC appointment within 1 month after AMI, two within 4 months and one within 5 months. Based on electronic health records, the median waiting period was 1 month (IQR: 1–2.5), with a range from 1 to 5 months.

Among participants who reported some form of PHC follow‐up (57.9%), several described it as limited in scope, often restricted to laboratory tests or prescription renewals rather than comprehensive care. As one participant explained, ‘Just one appointment at my initiative, and then only lab tests and exams’ (P1). Another echoed this sentiment: ‘It's just for lab tests and medication renewal’ (P7).

A few participants experienced more regular consultations, particularly when additional comorbidities were present. For instance, one said: ‘Now I have so‐called normal appointments. I already have a battery of tests to do… There should be two or three appointments annually if everything is fine’ (P3). Among those with diabetes, PHC follow‐up also included nursing appointments, reported by four participants, where care extended to blood pressure monitoring and foot checks: ‘I have appointments every 6 months with my doctor and nurse. My nurse measures blood pressure and checks my feet’ (P2).

Some participants (15.8%) indicated that they did not rely on PHC for follow‐up, because they were already receiving care through occupational health [‘I'm followed by occupational health at my work’ (P6)] or private providers [‘No follow‐up at the PHC, just private cardiology’ (P4)]. Three (15.8%) participants were referred to and completed phase II of the cardiac rehabilitation programme (CRP), after which they described PHC consultations as mostly administrative [‘Since I'm in the CRP, consultations at the PHC are only for lab tests and exams’ (P17). While one participant praised the engagement of CRP staff, they also noted the burden of frequent visits [‘In the CRP, they are exceptional because the professionals engage with people. But there are too many consultation appointments… I have to work’ (P5)].

Notably, the four (21.1%) participants who were rehospitalized within 8 months, reported receiving no PHC follow‐up after their initial hospital discharge, which may have contributed to their subsequent readmission.

Overall, PHC follow‐up after hospital discharge was characterised by reactive rather than proactive care, with most participants initiating appointments themselves and receiving limited, task‐oriented consultations focused on test requests or prescription renewals rather than holistic management. This pattern reflects a broader lack of systematic post‐discharge coordination and preventive engagement. Fragmented communication and unclear accountability between hospital and PHC teams further compounded this issue, as patients were often uncertain about which provider was responsible for their ongoing care, leading to gaps in follow‐up and continuity. Finally, limited patient engagement and shared decision‐making were evident in the absence of collaborative goal setting or structured discussions about recovery and self‐management, leaving patients to navigate care transitions largely on their own. This combination of passive follow‐up, unclear roles, and minimal patient involvement underscores a missed opportunity for PHC to function as an active partner in long‐term secondary prevention.

### 3.4 Hospital Follow‐Up

The median waiting time for the first hospital follow‐up appointment after discharge was 4 (IQR: 3–7) months, with considerable variation ranging from 1 to 13 months. Some participants expressed dissatisfaction with the length of time they had to wait, emphasising a desire for earlier and more frequent follow‐up. For example, one participant shared: ‘I felt that the appointment after the heart attack was too long after discharge!’ (P3); another noted ‘I only had a consultation 1 year after having a heart attack!’ (P8). Similarly one participant remarked, ‘The only thing I would change would be to be seen earlier and do more frequent exams. I was eager for the cardiology appointment’ (P16).

Of particular concern, the four (21.1%) participants who were rehospitalized within 8 months reported that they had not received any hospital follow‐up after discharge.

In summary, hospital follow‐up reflected a continuation of reactive rather than proactive care, with lengthy waiting times and limited mechanisms to ensure timely review after discharge. The absence of systematic coordination between hospital and PHC services illustrated fragmented communication and unclear accountability, leaving patients unsure who was responsible for monitoring their recovery or scheduling follow‐up appointments. This lack of structured continuity was particularly evident among the four participants who were rehospitalized without any interim follow‐up, underscoring system‐wide gaps in post‐discharge care. Together, these findings reveal a disjointed transition process in which delayed reviews, poor inter‐service communication, and minimal patient involvement compromise continuity and the potential for effective secondary prevention.

## 4 Discussion

The findings of this study offer valuable insights about the experiences of individuals who have had an AMI while navigating the Portuguese healthcare system, highlighting both strengths and critical areas for improvement. The findings are discussed below with existing literature and ESC recommendations, and in light of implications for practice and policy.

A total of 19 patient participants were recruited who were discharged during 2023 from a type A hospital (high level of technical expertise in cardiology). The median/mean age of the participants in this study was lower than that in the ESC EuroHeart registries (55 years *vs.* 68 years) [24]. However, this reflects the recruitment strategy, as our study recruited younger participants initially and was concluded once data saturation was achieved.

### 4.1 Preadmission Engagement With PHC

The analysis of pre‐existing cardiovascular risk factors highlights dyslipidemia (63.2%), hypertension (42.1%) and diabetes (31.6%), as the most prevalent conditions among participants. These findings are consistent with national and international epidemiological data, which indicate that these factors remain the dominant contributors to AMI [3, 25]. However, this study revealed important discrepancies between self‐reported information and electronic health records from PHC and hospital. In particular, smoking, coronary disease and dyslipidemia appeared less frequently documented in PHC records compared with patient self‐reports; for example, while 21.1% of participants identified themselves as smokers, only 5.3% had this diagnosis recorded in PHC. Similar inconsistencies of smoking documentation have been described in other studies, often attributed tolimitations in risk factor registration systems and inconsistent assessment of lifestyle behaviours [26].

Conversely, conditions such as hypertension, dyslipidemia, and diabetes were sometimes recorded in PHC at higher prevalence than participants themselves acknowledged. Rather than interpreting these discrepancies as direct evidence of under or over‐reporting, they are best understood as illustrations of communication and documentation gaps between patients and healthcare providers. Such differences may reflect variations in health literacy, information exchange, or record‐keeping practices rather than a lack of patient awareness alone. Comparable patterns have beenreported in previous European studies, where challenges in patient–provider communication and health record accuracy have been identified as barriers to effective chronic disease management [27, 28].

A particularly concerning finding is the low level of reported preventive engagement with PHC: only 36.8% of participants described regular follow‐up before their AMI, mostly those with diabetes or hypertension. Even among participants with established coronary disease, only one reported structured cardiovascular risk management. This suggests a shortfall in proactive risk surveillance and management within PHC settings [25]. This pattern of sporadic and reactive use of PHC mirrors evidence from Portuguese [29] and European studies showing that individuals often access PHC for acute issues or administrative purposes rather than continuous preventive monitoring [30].

Together, these findings reinforce the idea that PHC in Portugal may not be fully capitalising on its role in cardiovascular risk prevention, with cardiovascular risk factors remaining underdiagnosed or suboptimally managed [31, 32]. The under‐identification of smoking, the gaps in patient awareness of their own conditions and the low engagement in preventive care represent missed opportunities for early intervention.

Strengthening systematic risk factor screening, improving patient education, and enhancing continuity of care have been repeatedly recommended in the literature [3, 25], and our results provide further evidence supporting the urgent need for these measures in the Portuguese healthcare context.

### 4.2 Hospital Discharge

While many participants acknowledged receiving general guidance post‐discharge (e.g. cholesterol, blood glucose levels, exercise and smoking cessation), few reported being given specific, measurable, or documented goals. Instead, advice was often broad and nonspecific, leaving patients with only a vague sense of what they should do, without clear parameters of success. Evidence suggests that such vague discharge recommendations hinder long‐term adherence and limit patient empowerment [33, 34]. For example, Albrecht and collaborators [35], found that gaps affected 5% of patients for follow‐up appointments, 27% for medications, 48% for exercise and 50% for diet recommendations, with risk factors for misunderstanding including older age, social isolation and depression. These results mirror our results, where patients frequently described vague instructions and a lack of clear goals, suggesting that both the content and delivery of discharge information may contribute to confusion, limited engagement, and suboptimal self‐management.

These observations underscorea critical shortfall in personalised discharge planning and collaborative goal setting. Establishing clear, specific and achievable treatment targets is essential to support long‐term patient adherence and outcomes [2, 3]. Interpreting these findings through the lens of Goal Theory provides a useful framework for understanding why this gap persists. As Park [36, 37] notes, goals that are well‐defined, personally meaningful and attainable are central to sustaining motivation andadherencein chronic illness management The lack of such structured goal setting in our participants’ experiences helps explain the difficulty many faced in maintaining engagement after discharge.

Complementarily, Meleis’ Transitions Theory [38] helps interpret the emotional and behavioural dimension of these experiences. An AMI represents a profound life transition: a critical event that disrupts daily life and requires adaptation to a new health reality. According to the theory, successful transitions depend on awareness, engagement, and capacity for change. Our findings suggest that while the AMI experience heightened awareness for many patients, the absence of structured guidance and shared goal‐setting limited their ability to translate that awareness into sustained behaviour change. Together, these frameworks highlight that post‐discharge recovery is not solely a matter of providing information but of facilitating an adaptive process. Nursing and multidisciplinary interventions play a key role in supporting patients through this transition, helping them define meaningful goals, build confidence, and adopt long‐term health‐promoting behaviours. An effective way to address this shortfall involves shared decision‐making (SDM), in which patients and healthcare providers collaborate to establish meaningful, achievable, and personalised goals. The 2023 ESC Guidelines for the management of acute coronary syndromes explicitly recommend SDM (Recommendation Class I, Level B) [3]. However, our findings suggest that shared decision‐making is not yet embedded as routine practice in Portuguese cardiac care, which resonates with recent analyses indicating limited clinician adoption of SDM [39, 40]. Furthermore, a population‐based survey using the Problem‐Solving Decision‐Making Scale showed that two‐thirds of Portuguese individuals prefer doctors to take the lead in serious health decisions, with few favouring shared decision‐making even in less critical scenarios [41]. Such cultural and systemic factors may partly explain why goal‐setting remains vague, clinician‐driven and poorly recalled by patients.

Patient‐Reported Outcome Measures (PROM) and Patient‐Reported Experience Measures (PREM) could play a pivotal role in bridging this gap. Although participants did not report their use, it remains unclear whether they were ever applied during their care. Their absence from patients’ narratives, however, highlights a potential missed opportunity to systematically capture patient‐centred outcomes and experiences. Supporting this, the Organisation for Economic Co‐operation and Development (OECD) PaRIS survey reveals that people with chronic conditions in Portugal report notably poorer outcomes and experiences compared to the OECD average. Specifically, only 57% report good physical health, 67% report good mental health, 49% experience good care coordination and 77% experience person‐centred care, each well below the OECD PaRIS average. These deficits underscore the lack of structured PROMs and PREMs in routine practice and underscore the importance of incorporating such tools into discharge planning [42].

By embedding PROMs and PREMs within a shared decision‐making framework, goal‐setting could become more structured, measurable, and patient‐centred. Evidence consistently shows that structured goal‐setting, supported by SDM and informed by PROMs/PREMs, improves patient engagement, adherence and long‐term outcomes [2, 3, 43].

Medication counselling also revealed important gaps. Although 84.2% participants recalled receiving some explanation about medication, only a few recalled being informed about potential side effects, drug interactions or long‐term adherence strategies. Both interviews and analysis of healthcare records confirmed that communication tended to focus on dosage and administration, while risks were rarely discussed proactively. Similar patterns have been observed in other European contexts (Central Europe), and the evidence shows that inadequate medication counselling can contribute to nonadherence and higher readmission rates, which suggests a potential risk in this Portuguese setting [44, 45, 46]. In our study, two participants described readmission following unsupervised discontinuation and subsequent adverse events, suggesting that, although infrequent, such situations may illustrate a potential risk associated with lack of follow‐up. According to ESC recommendations, upon hospital discharge, patients should receive a copy of their discharge summary, including therapeutic targets and information on follow‐up care. Patient information materials, such as leaflets, can be helpful if written in plain language, avoiding medical jargon and abbreviations, highlighting key points, and using large fonts and illustrations. All aspects of self‐care should be reviewed with the patient before discharge to enhance understanding, improve medication adherence, and reduce the risk of complications, including readmission. Given the variability in health literacy, information should be delivered in manageable portions, with comprehension checked regularly, using approaches such as the teach‐back method or motivational interviewing [3]. International studies confirm that simplified discharge letters [33, 44] and teach‐back strategies [3, 47] significantly improve patient comprehension and safety after discharge. Within the Portuguese healthcare system, nurses [48] and pharmacists [49] are particularly well‐positioned to reinforce medication adherence and education.

Despite these shortcomings, participants expressed high satisfaction with in‐hospital care, rating it 10 (IQR:9.5–10) out of 10. However, such high satisfaction contrasts with reports of insufficient individualised discharge planning and weak long‐term care coordination. Similar discrepancies have been observed in other cardiac care settings, where positive perceptions of acute‐phase satisfaction mask systemic issues in continuity of care and patient empowerment [50].

Our findings suggest that the current model of discharge communication represents a missed opportunity to align care with best practice recommendations. Embedding SDM and systematically integrating PROMs and PREMs into clinical routines could help transform vague clinician‐driven advice into actionable, collaborative treatment plans that foster meaningful behavioural change, strengthen adherence and ultimately improve long‐term outcomes.

Taken together, the triangulated findings from interviews and discharge documents reveal a consistent pattern: treatment goals are communicated to patients at discharge, but rarely negotiated with them. As a result, goals tend to be vague, lack measurable targets, and are often poorly recalled or understood. They are also not systematically recorded in discharge notes, which patients are neither encouraged to read nor supported to fully understand. This underscores the absence of shared decision‐making. Recommendations are delivered by health professionals, yet there is little evidence that patients' preferences, capacities, or willingness to engage are taken into account. In practice, this disconnect means that what physicians prescribe and what patients are prepared or able to implement may diverge, thereby limiting the potential for meaningful behaviour change and effective long‐term adherence.

### 4.3 PHC Follow‐Up

PHC follow‐up after discharge showed substantial variability. Notably, 84.2% of follow‐up encounters were initiated by patients themselves. This pattern suggests that continuity of care is overly dependent on patient initiative, an approach that risks leaving behind those with limited self‐management or lower health literacy. Such reliance on patient‐driven follow‐up echoes findings from other chronic disease management studies (i.e. patients receiving PHC follow‐up after hospital discharge for heart failure experienced a 20% decrease in 30‐day all‐cause readmissions), and evidence has shown that insufficient proactive engagement from PHC has been linked to poorer long‐term outcomes [51]. Although 57.9% of participants reported receiving some form of PHC follow‐up, these interactions were often limited to lab tests, medication renewals, and general exams, with few opportunities for deeper clinical assessments or personalised health coaching. This pattern suggests that follow‐up care was primarily reactive rather than preventive in scope.

Interestingly, studies such as that ofTavares and collaborators [52] have highlighted a gap between patients' expectations and the preventive measures typically emphasised in PHC. In that study, patients reported valuing comprehensive assessments, including cardiac and pulmonary auscultation (82.6%), evaluation of physical activity (74.7%) and tobacco cessation counselling (72.9%), more highly than physicians anticipated. While our findings do not directly measure such preferences, the contrast may reflect broader system‐level constraints (e.g., limited consultation time, staffing, or resource availability) that restrict opportunities for more proactive, preventive engagement. Thus, rather than a simple mismatch of expectations, the limited scope of post‐MI follow‐up in our study likely reflects the structural challenges of implementing patient‐centred preventive care in PHC settings.

The limited provision of nursing appointments in PHC, mostly confined to diabetic patients, may represent a missed opportunity for broader risk factor management and patient education. In contrast, international evidence indicates that nurse‐led interventions can improve risk factor control, adherence and overall clinical outcomes [53, 54, 55]. A multicenter quasi‐experimental study of 212 post‐AMI patients across 40 PHC settings showed that an intensive, nurse‐led follow‐up over 12–18 months significantly enhanced self‐management and adherence behaviours, including diet, physical activity and medication use. The authors conclude that structured nurse‐led interventions 1 month after AMI can boost patient self‐efficacy and long‐term adherence, particularly when combined with family support [53].

Moreover, PHC teams are not systematically notified when a patient is discharged from the hospital. This lack of formal communication represents a critical gap, as it undermines the ability of PHC providers to proactively engage patients, ensure timely follow‐up and coordinate secondary prevention measures.

Regarding PHC follow‐up appointments after discharge, 57.9% of participants reported having such consultations, with a median waiting period of 1 month (IQR: 1–2.5), ranging between 1 and 5 months. Following hospital discharge post‐AMI, studies reported that the average time to review lipid‐lowering therapy ranged from 6.2 weeks in Italy to 10.5 weeks in the UK [56]. This aligns with the 2019 ESC Guidelines for the Diagnosis and Management of Chronic Coronary Syndromes [57], which advocate for a timely review of the patient's response to medical therapies, ideally within 2–4 weeks after drug initiation (Recommendation Class I, Level C).

Overall satisfaction with PHC was lower than with hospital services (mean 7.92 ± 2.02 out of 10). The lack of clearly defined objectives or specific goals established with patients at the time of discharge, along with the failure to document them in the discharge summary, has been identified as a significant barrier to effective post‐AMI care in PHC settings [2]. This aligns with a recent study with cardiology nurses which reported that limited time for patient education, insufficient training in engagement strategies, lengthy and complex discharge documents, poor communication with PHC, lack of medication reconciliation, and unfamiliarity with referral systems all contribute to unclear care plans, leaving patients uncertain about treatment and undermining adherence [58].

### 4.4 Hospital Follow‐Up

In the current study, participants reported that the first hospital follow‐up appointment after discharge typically occurred around 5 months post‐AMI, with considerable variation ranging from 1 to 13 months. Many expressed dissatisfaction with these long waiting times and a desire for earlier and more frequent consultations. These findings align with previous research in Portugal, which found that post‐discharge follow‐up generally began with a first cardiology visit around 3 months after discharge, followed by visits every 6 months for 1–2 years, although schedules were highly variable and sometimes inconsistent between professionals within the same hospital [10]. The timeline for the first hospital follow‐up appointment after discharge in our study exceeds the recommendations of the 2018 ESC/EACTS Guidelines on Myocardial Revascularization, which advise re‐evaluating patients at 3 months postprocedure and at least annually thereafter (Recommendation Class I, Level C) [59]. Resource constraints in the Portuguese healthcare system, as highlighted by Fontes‐Carvalho and collaborators, may contribute to these delays, limiting the capacity of hospitals to ensure timely follow‐up [10].

Furthermore, 15.8% of participants were referred to and completed phase II of the CRP, notably higher than the national average (9.3%) [60]. This suggests that access to structured rehabilitation was comparatively enhanced in this setting, reflecting the hospital's higher‐level resources and capabilities.

## 5 Implications for Practice

Building on these findings and considering the gaps identified in comparison with ESC guideline recommendations, several practical implications emerge to strengthen post‐AMI care, as summarised in Table 2. These proposed measures address key transition points across hospital discharge, PHC follow‐up, and hospital follow‐up to improve coordination, enhance patient engagement, and promote timely secondary prevention.

**Table 2:** Recommendations for practice.

**Hospital discharge**	Establish formal collaboration protocols between hospitals and PHC; Develop a patient leaflet outlining the care pathway, follow‐up timelines, ESC‐recommended targets, and self‐care guidance; Provide training for healthcare professionals about PREMs and PROMs, to incorporate into routine clinical practice; Improve discharge documentation by including clearly negotiated therapeutic goals with patients; Provide training for healthcare professionals about the electronic health record to notify PHC about hospital discharges; Promote the use of the app SNS24 to empower patients in recording and monitoring health data; Encourage patients to read and understand their discharge letters, ensuring translation into plain language when needed;
**PHC follow‐up**	Schedule a follow‐up consultation within 1 month of hospital discharge; Introduce nurse‐led consultations for post‐AMI monitoring in PHC.
**Hospital follow‐up**	Develop and implement criteria to prioritise earlier follow‐up appointments for higher‐risk patients; Introduce nurse‐led telephone consultations for post‐AMI monitoring 1 month after discharge.

### 5.1 Strengths and Limitations

A key strength of this study is the inclusion of patient voices, which provide nuanced insights into barriers and facilitators of continuity of care, extending beyond what quantitative studies can capture. Furthermore, triangulation with electronic health records allowed the identification of gaps that could not be captured through self‐reported data alone, such as pre‐existing cardiovascular risk factors and the timing of follow‐up appointments in both PHC and hospital settings. Finally, validation of the findings by a multidisciplinary group of researchers, including professionals from different levels of care and patient representatives, was crucial to ensure that all perspectives were adequately reflected.

Nevertheless, several limitations must be acknowledged. First, participants were not explicitly asked about PROMs/PREMs or quality‐of‐life consultations, which may have limited the depth of findings regarding patient‐reported outcomes. Second, although the sample size was adequate for qualitative analysis, it may not fully reflect the diversity of experiences across all Portuguese regions. Third, the staged purposive sampling strategy we employed (beginning with younger patients with fewer comorbidities and more linear trajectories before progressing to more complex cases) was valuable in building analytical clarity and monitoring thematic saturation. However, this approach may have delayed the inclusion of perspectives from patients with more complex care pathways, potentially influencing the range of experiences captured. Fourth, the cross‐sectional nature of the interviews captures patient perceptions at a single point in time, without capturing how these experiences may evolve longitudinally. Fifth, the study was conducted in a Type A Hospital, which may limit generalisability to other healthcare settings. Nonetheless, it remains important to identify local problems to adapt the proposed improvements to the specific context and resources of each Local Health Unit to ensure feasibility and relevance.

Additionally, the potential influence of the lead researcher's professional role as a cardiology nurse must be considered. Although the nurse had no prior involvement in participants' direct care, disclosure of this professional background may have influenced how participants responded during interviews and could have introduced unconscious bias in the analysis. This potential effect should be acknowledged when interpreting the findings.

Furthermore, while the research materials were reviewed by the multidisciplinary team, including patient representatives, they were not formally piloted with participants before data collection. Piloting could have ensured that the language and phrasing were fully appropriate for patient understanding. The absence of a pilot represents a limitation, although involvement of patient representatives in reviewing materials partially mitigates this concern. The full interview schedule is provided as a supporting file to enhance transparency and reproducibility.

Finally, we acknowledge that presenting the visual map of findings to participants for reflection could have strengthened analytical validation, allowing participants to confirm that the identified themes accurately reflected their experiences. In addition, the study benefited from the inclusion of two patient representatives as active members of the research team. These lay members contributed to the development of study materials, reviewed preliminary analyses, and participated in refining the visual journey map, ensuring that interpretations remained patient‐centred. Their involvement represents a key strength of the study, as it helped ground the research in real‐world patient perspectives and enhanced the credibility and relevance of the findings, aligning it more closely with Patient and Public Involvement and Engagement (PPIE) principles.

Despite these limitations, this study makes an important contribution to the evidence base by identifying critical gaps in preventive care, discharge planning, and post‐discharge continuity, and by situating Portuguese experiences within broader European and global patterns.

## 6 Conclusion

This study provides novel insights into patient experiences of post‐AMI care in Portugal, highlighting critical gaps in discharge communication, shared decision‐making, coordination with PHC, and timely follow‐up. Despite high levels of satisfaction with in‐hospital treatment, patients frequently reported vague discharge goals, limited medication counselling, and fragmented continuity of care. Preventive engagement with PHC was often reactive rather than proactive, and long waiting times for hospital follow‐up appointments exceeded ESC guideline recommendations, exposing patients to avoidable risks.

By incorporating patient narratives, triangulated with electronic health records and validated by a multidisciplinary team including patient representatives, this study underscores the importance of adopting a patient‐centred approach that values both clinical outcomes and lived experiences. Such an approach is essential to strengthen continuity of care, promote adherence, and improve long‐term outcomes after AMI.

The findings directly inform practical strategies to bridge the gap between evidence and practice, including the introduction of structured discharge protocols with negotiated therapeutic targets, enhanced communication about medication and side effects, systematic use of PROMs and PREMs, earlier and nurse‐led follow‐up in PHC, and clear coordination mechanisms between hospitals and PHC.

Ultimately, strengthening post‐AMI care in Portugal requires a shift towards integrated, equitable and person‐centred cardiovascular services. Insights from this study provide a valuable foundation for co‐designing and piloting patient‐centred interventions within the Cardiac Integrated Care project, with the potential to significantly enhance recovery, reduce readmissions, and promote healthier lives for individuals living with cardiovascular disease.

## Author Contributions

**Filipa Homem:** conceptualisation, writing – original draft, writing – review and editing, formal analysis and validation. **Andreia Gomes:** conceptualisation, validation, writing – review and editing and formal analysis. **Helena Martins:** conceptualisation, validation, writing – review and editing and formal analysis. **Ana Caetano:** conceptualisation, writing – review and editing. **Maurício Alves:** conceptualisation, writing – review and editing. **Luís Rodrigues:** conceptualisation, writing – review and editing. **Mariana Simões:** conceptualisation, writing – review and editing. **Sofia Martinho:** conceptualisation, writing – review and editing. **Sílvia Monteiro:** conceptualisation, writing – review and editing. **Catarina Veiga:** conceptualisation, writing – review and editing. **Emília Correia:** conceptualisation, writing – review and editing. **Dário Lemos:** conceptualisation, writing – review and editing. **Verónica Coutinho:** conceptualisation, writing – review and editing. **António Amaral:** conceptualisation, writing – review and editing.

## Ethics Statement

Ethical approval was obtained from the ULS Coimbra Ethics Committee (2024‐1‐ESI. SF) on 8 March 2024.

## Conflicts of Interest

The authors declare no conflicts of interest.

## Supporting information

TELEPHONE INTERVIEW SCRIPT.

## Data Availability

The authors confirm that the supplementary data are available from the corresponding author upon reasonable request.
