# Supplementary material for: Enhancing Post‐Discharge Care for People Who Have Had an Acute Myocardial Infarction in Portugal: Insights From Patient Journey Mapping
Source: Health Expect. 2025 Dec 19;28(6):e70521. doi: 10.1111/hex.70521 (PMC12716438; doi:10.1111/hex.70521)
Supplement: Supplementary file 1 — TELEPHONE INTERVIEW SCRIPT. [file HEX-28-e70521-s001.docx]

**TELEPHONE INTERVIEW SCRIPT**

Good morning, my name is FH, and I am a cardiology nurse at ULSC. I am calling because you were recently discharged from our hospital, and we would like to understand your experience with your hospital stay and post-discharge follow-up to help us improve the quality of care we provide.

The interview should take no more than 20 minutes. Would you like to participate? Is this a convenient time?

During this interview, I will ask you questions about your hospitalization and your follow-up care after discharge. Please answer honestly based on your experiences. Your responses will not affect your current or future medical care.

I would like to inform you that this call will be recorded for content analysis only, and your identity will remain anonymous. The data will be anonymized and may be used for scientific purposes. You can withdraw at any time, and your information will be removed. This consent covers both participation in the interview and authorization to access relevant clinical data from your electronic health record. Do you consent to participate?

**First, regarding your hospitalization:**

- How many days were you hospitalized? Did you feel this was too short, adequate, or too long?
- Do you know the reason for your hospitalization?
- Was this your first hospital stay, or have you been hospitalized in cardiology before? If so, when, for how long, and for what reason?
- Before hospitalization, what was your lifestyle like? Did you have any health problems, and were you taking any medications?
- Before your hospitalization, did you have regular appointments at your primary health care center (PHC)? Did the healthcare team ask about your medications or lifestyle?

**At discharge**

- Were any specific treatment goals discussed with you, such as improving cholesterol, adjusting medication, or increasing physical activity?
- Did you receive a complete list of all your medications, including non-cardiology drugs, and were you informed about their purpose and potential side effects?
- Were you informed about follow-up care, including which signs and symptoms to monitor and which risk factors to modify?
- Do you feel you had a clear understanding of your health condition and the factors that could improve or worsen it?
- Did you have enough time and opportunity to ask questions?
- After leaving the hospital, did you feel confident that you could manage your health? What challenges did you experience after discharge?

Overall, on a scale from 0 to 10, where 0 is not at all satisfied and 10 is extremely satisfied, how would you rate the quality of care you received during your hospital stay? Would you recommend this service to others?

**Next, regarding post-discharge follow-up**:

- Do you usually follow up at your PHC, either routinely or only when sick?
- Were you contacted by your PHC after discharge?
- Did you schedule a follow-up appointment yourself at your PHC or in the private sector?
- Did you attend a follow-up appointment at your PHC?
- During these visits, did anyone discuss your lifestyle or treatment goals with you?
- Were your prescribed medications reviewed? Do you take them as prescribed? Do you have any questions or experience side effects?

Overall, on a scale from 0 to 10, how would you rate the quality of care provided by your PHC? Would you recommend this center to others?

**Hospital follow-up:**

- After your heart attack, did you have a follow-up appointment at the hospital? If so, when and how was it?

Thank you very much for your time. Your responses are extremely valuable and will help us improve the quality of care we provide.
